# CD38-targeted therapy with daratumumab reduces autoantibody levels in multiple myeloma patients

**DOI:** 10.1016/j.jtauto.2019.100022

**Published:** 2019-11-11

**Authors:** Kristine A. Frerichs, Christie P.M. Verkleij, Patricia W.C. Bosman, Sonja Zweegman, Henny Otten, Niels W.C.J. van de Donk

**Affiliations:** a Amsterdam UMC, Vrije Universiteit Amsterdam, Department of Hematology, Cancer Center Amsterdam, Amsterdam, the Netherlands; b University Medical Center Utrecht, Laboratory of Translational Immunology, Utrecht, the Netherlands

**Keywords:** Antibody-mediated autoimmune disease, Daratumumab, Autoreactive, Plasma cells, CD38

## Abstract

Autoantibody-producing plasma cells are frequently resistant to conventional immunosuppressive treatments and B-cell depletion therapy. As a result of this resistance, autoreactive plasma cells survive conventional therapy, resulting in persistent autoantibody production and inflammation. CD38 is highly and uniformly expressed on normal and malignant plasma cells. Daratumumab is the first in class CD38-targeting monoclonal antibody approved for the treatment of multiple myeloma (MM). To evaluate the potential activity of daratumumab in antibody-mediated autoimmune disorders by targeting autoantibody-producing plasma cells, we evaluated serum levels of autoantibodies in MM patients during daratumumab treatment. We found that 6 out of 41 (15%) had detectable autoantibodies before initiation of daratumumab therapy, and that these autoantibodies rapidly disappeared in 5 out of 6 patients during daratumumab treatment. Our data provide support for the evaluation of daratumumab in patients with autoantibody-dependent autoimmune disorders.

## Highlights

• Autoantibody-producing plasma cells express CD38.
• The CD38-targeting antibody daratumumab reduces autoantibody levels.
• Our data provide support for the evaluation of daratumumab in antibody-mediated autoimmune disease.

## 1 Introduction

Autoantibodies, produced by plasma cells (PCs), contribute to the pathogenesis of antibody-mediated autoimmune diseases such as systemic lupus erythematosus (SLE), rheumatoid arthritis (RA), anti-neutrophil cytoplasmic antibodies (ANCA)-associated vasculitis, myasthenia gravis, and autoimmune cytopenias. These autoantibodies are either directed against cells expressing the target antigen, or cause tissue damage via formation of immune complexes. Autoantibody-producing PCs are found in bone marrow, but are also abundant in inflamed tissues, for instance in synovial biopsies obtained from RA patients or in nephritic kidneys from SLE patients, leading to local production of auto-antibodies [1,2]. These autoreactive PCs are typically long-lived and are frequently resistant to conventional immunosuppressive treatments, as well as B-cell depletion therapy with CD20 or CD22-targeting agents [3,4]. As a result of this resistance, autoreactive PCs survive conventional therapy, which leads to persistent production of autoantibodies that maintain autoimmunity and auto-inflammatory processes. There is an unmet need for novel therapeutic interventions in antibody-mediated autoimmune diseases, especially for those with disease resistant to conventional therapies or presenting with aggressive features. In this respect, specific targeting of long-lived PCs represents a novel treatment approach.

CD38 is highly and homogeneously expressed on normal PCs, as well as malignant PCs from multiple myeloma (MM) patients [5]. Daratumumab is the first in class CD38-targeting antibody, which has single agent activity in MM. Based on its high activity and favorable toxicity profile, daratumumab is currently approved as monotherapy, as well as in combination with several standards of care in MM [[6], [7], [8]].

Next to the elimination of malignant PCs, we have previously shown that daratumumab reduces the frequency of normal PCs in MM patients, which resulted in decreased levels of circulating polyclonal immunoglobulins [9]. We therefore hypothesized that daratumumab is also capable of depleting autoantibody-producing, non-malignant PCs. In the current study, the impact of daratumumab on autoantibody levels in MM patients was used as a surrogate parameter for its effect on autoreactive PCs.

## 2 Materials and methods

### 2.1 Patient samples

We evaluated the presence of autoantibodies in residual serum samples obtained from 41 extensively pretreated MM patients, who received daratumumab monotherapy in the DARA-ATRA study (NCT02751255; n = 29), or daratumumab combined with the programmed death-1 (PD-1) inhibitor nivolumab in the NIVO-DARA study (NCT03184194; n = 12). All patients were treated at the Amsterdam University Medical Center from 2016 to 2019. Daratumumab was administered as an intravenous infusion at a dose of 16 mg/kg (8-times once weekly, 8-times every 2 weeks, and then every 4 weeks until disease progression). Nivolumab was administered as an intravenous infusion at a dose of 240 mg twice weekly for the first 24 weeks, and 480 mg every 4 weeks hereafter until disease progression. The study site ethics committee approved the protocols, which were conducted according to the principles of the Declaration of Helsinki, the International Conference on Harmonization and the Guidelines for Good Clinical Practice. All patients gave written informed consent.

### 2.2 Autoantibody assessments

Samples obtained prior to the initiation of daratumumab and/or nivolumab treatment (baseline) were analyzed for the presence of antinuclear antibodies (ANA), anti-neutrophil cytoplasmic antibodies (ANCA), and rheumatoid factor (RF, an autoantibody directed against the Fc portion of IgG). If ANA were detected by using the indirect immunofluorescence assay, additional testing for antigen-specificity was performed by measuring anti-double stranded DNA (anti-dsDNA), anti-Sjögren’s syndrome-related antigen A and B (anti–SS–A; anti–SS–B), anti-Smith antigen (anti-Sm), anti-U1 ribonucleoprotein (anti-U1RNP), anti-Centromere protein B, anti Sclerostin-70 (anti-Scl70) and anti-Jo-1 antibodies using Enzyme-Linked Immunosorbent Assay (ELISA). If ANCA were detected by using the indirect immunofluorescence assay, additional analyses were performed to assess the presence of myeloperoxidase (MPO) or proteinase-3 (PR-3) ANCA using ELISA. RF was detected using a fluorescence enzyme immunoassay (upper limit of normal <5 IU/mL). If an autoantibody was present at baseline, we serially assessed autoantibody levels at approximately 4–8 week intervals. Anti-tumor response was determined every 4 weeks by quantification of M-protein and/or free-light chain levels.

### 2.3 Statistics

ANA titers were qualified as negative, weakly positive, positive or strongly positive. ANCA titers were quantified as negative, 1:40, 1:80, or 1:160. RF antibody titers were quantified in IU/mL. Graphs were constructed using Graphpad Prism version 7.

## 3 Results

### 3.1 Autoantibodies are detected in 15% of heavily pretreated MM patients prior to initiation of daratumumab treatment

**Fig. 1:**
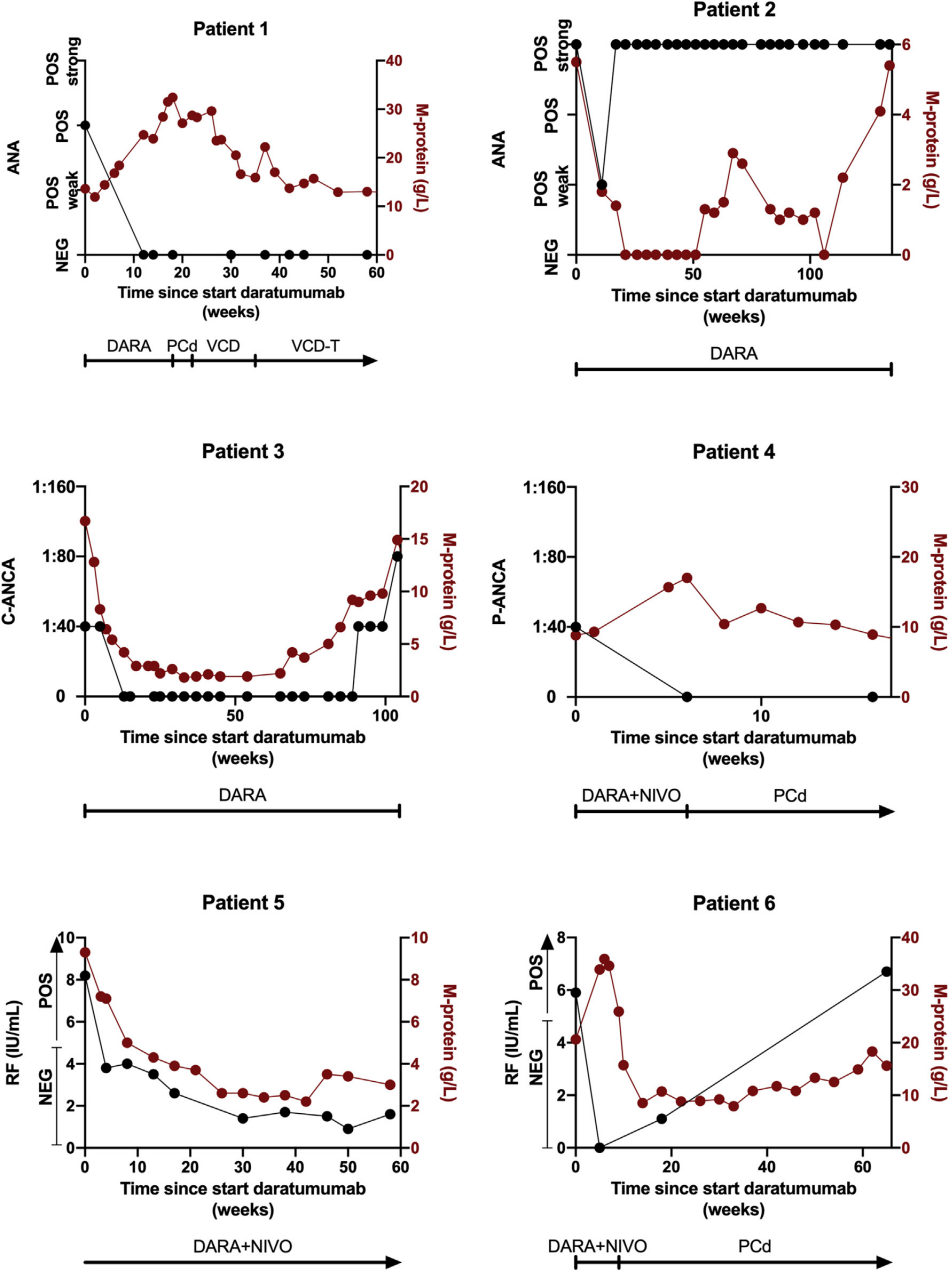
Daratumumab reduces autoantibody levels in multiple myeloma patients. Six out of 41 evaluated patients had positive autoantibody titers prior to initiation of treatment with daratumumab monotherapy (patients 1, 2, and 3), or daratumumab plus nivolumab (patients 4, 5, and 6). Daratumumab was administered as an intravenous infusion at a dose of 16 mg/kg (8-times once weekly, 8-times biweekly, and then every 4 weeks until disease progression). Nivolumab was administered as an intravenous infusion at a dose of 240 mg biweekly for the first 24 weeks, and 480 mg every 4 weeks hereafter until disease progression. The black lines show the effect of daratumumab on antinuclear antibodies (ANA; patients 1 and 2), anti-neutrophil cytoplasmic antibodies (ANCA; patients 3 [C-ANCA] and 4 [P-ANCA]), and rheumatoid factor (RF; patients 5 and 6). Anti-tumor response, as determined by evaluation of M-protein levels, is also demonstrated for each patient in the graphs (red lines). If patients developed disease progression during daratumumab treatment and received a subsequent line of anti-MM therapy, autoantibody titers and anti-tumor responses to subsequent therapies are also shown. Abbreviations: DARA, daratumumab; PCd, pomalidomide-cyclophosphamide-dexamethasone; VCD, bortezomib-cyclophosphamide-dexamethasone; VCD-T, bortezomib-cyclophosphamide-dexamethasone-thalidomide; NIVO, nivolumab.

Six out of 41 (15%) patients tested positive for an autoantibody (Fig. 1). Three patients were treated with daratumumab monotherapy, and three patients with daratumumab and nivolumab. There were no patients with positivity for more than one autoantibody. None of these patients had clinical manifestations of autoimmune disease.

### 3.2 During daratumumab treatment, ANA levels are reduced in one patient, but remain detectable in the second patient

The autoantibody screening revealed a positive ANA with nucleolar staining pattern in 2 patients. Additional testing for antigen-specificity (anti-dsDNA, anti–SS–A, anti–SS–B, anti-Sm, anti-U1RNP, anti-Centromere protein B, anti-Scl70 and anti-Jo-1 antibodies) was negative in both patients. In patient 1, the ANA test became negative after initiation of daratumumab monotherapy, which persisted >40 weeks after daratumumab was discontinued due to disease progression. Although patient 2 achieved a complete response of the malignant PC disorder, the ANA test remained positive.

### 3.3 During daratumumab treatment, ANCA disappear in two patients

The indirect immunofluorescence assay demonstrated the presence of ANCA in two different patients: one with a cytoplasmic staining pattern (C-ANCA; patient 3), and one with perinuclear pattern (P-ANCA; patient 4). The MPO- and PR3-ANCA ELISAs were negative. In both patients, ANCA disappeared after initiation of daratumumab treatment. In patient 3, ANCA became detectable again at the same time of MM disease progression. In contrast, although follow-up was short, the ANCA test remained negative in patient 4 after discontinuation of daratumumab treatment due to MM disease progression.

### 3.4 During daratumumab treatment, RF titers are reduced in two patients

Finally, RF was detected in 2 other patients using a fluorescence enzyme immunoassay. Four weeks after the first dose of daratumumab, RF titers became negative in both patients. The RF titer decreased from 8.2  at baseline to a nadir of 0.9 IU/mL in patient 5. At the time of last follow-up, there was continued control of MM and the RF titer remained negative. In patient 6, RF titer decreased from 5.9 to 0.0 IU/mL. This patient developed progression of MM during daratumumab therapy. Although the RF titer remained suppressed 9 weeks after the last daratumumab infusion, a follow-up measurement 56 weeks later showed that the RF titer had returned to baseline levels.

## 4 Discussion

This proof of concept demonstrates that daratumumab treatment results in a rapid and sustained reduction of autoantibody titers in the majority of patients (5 out of 6; 83%), indicating that the drug effectively depletes autoreactive PCs. Although several studies have demonstrated that reduction of autoantibody levels is associated with clinical improvement and reappearance reflects relapse, the effect of daratumumab on clinical parameters in patients with autoimmune diseases has yet to be investigated in prospective clinical studies. However, additional support for the role of CD38-targeted therapy in autoimmune diseases comes from 2 case reports that recently showed the potency of daratumumab in the treatment of post-transplant autoimmune hemolytic anemia refractory to established options, and treatment-refractory pure red blood cell aplasia after ABO-mismatched allogeneic stem cell transplantation [10,11]. This approach is further supported by a recent study which showed that daratumumab depleted PCs and plasmablasts in blood samples obtained from SLE and RA patients in an *ex vivo* experimental setting [1]. Finally, we have previously shown that daratumumab may also be effective in the treatment of severe allergies by eliminating IgE-producing PCs which resulted in decreased levels of circulating total- and antigen specific IgE [12].

Importantly, 3 of our patients received daratumumab combined with the PD-1 inhibitor nivolumab. It is unlikely that the reduced autoantibody levels are the result of nivolumab treatment, since it has T-cell stimulatory effects, but no direct PC activity.

Daratumumab is well tolerated, with infusion-related reactions as the most common adverse event [5]. Infectious complications may be a concern when specifically targeting PCs. However, we have recently shown that a fraction of normal PCs persists during daratumumab treatment, and that daratumumab-treated MM patients produce protective antibody titers following vaccination, which is in contrast to patients treated with B-cell depleting regimens [9]. Importantly, additional studies are required to investigate whether adverse events and long-term effects in patients with autoimmune diseases are different from those observed in MM patients.

Other strategies of targeting PCs in patients with autoimmune diseases include the use of different anti-MM agents such as the proteasome inhibitor bortezomib. In contrast to daratumumab, bortezomib is not PC-specific and induces neuropathy in the majority of patients. Antibodies that block B-cell activating factor (BAFF) or a proliferation inducing ligand (APRIL), and thereby prevent the differentiation of B cells to PCs, have also shown efficacy in autoimmune diseases. Since autoantibodies recurred during follow-up in 2 out of our 6 patients, and autoantibody titers were not affected by daratumumab in another patient, the combination of daratumumab with other PC-directed therapies may be necessary to completely eliminate autoreactive PCs.

## 5 Conclusion

We show that daratumumab is capable of depleting autoantibody-producing PCs. Our study provides further support for the evaluation of daratumumab in patients with RA and SLE or other types of autoantibody-dependent autoimmune disorders.

## Disclosure of conflicts of interest

SZ and ND received research support from 10.13039/100005205Janssen Research and Development and BMS, and served in advisory boards from 10.13039/100008897Janssen Pharmaceuticals and BMS. All other authors have no conflicts to disclose.

## Funding sources

The clinical studies were supported by 10.13039/100008897Janssen Pharmaceuticals and 10.13039/100002491BMS, United States.

## Authorship contributions

KF, CV, SZ, PB, and ND provided patient materials; KF and ND designed the study, analyzed and interpreted the results, and wrote the first draft of the manuscript; all authors helped critically review the manuscript and checked the final version of it.

## References

[1] Cole S., Walsh A., Yin X., Wechalekar M.D., Smith M.D., Proudman S.M. (2018). Integrative analysis reveals CD38 as a therapeutic target for plasma cell-rich pre-disease and established rheumatoid arthritis and systemic lupus erythematosus. Arthritis Res. Ther..

[2] Starke C., Frey S., Wellmann U., Urbonaviciute V., Herrmann M., Amann K. (2011). High frequency of autoantibody-secreting cells and long-lived plasma cells within inflamed kidneys of NZB/W F1 lupus mice. Eur. J. Immunol..

[3] Hiepe F., Radbruch A. (2016). Plasma cells as an innovative target in autoimmune disease with renal manifestations. Nat. Rev. Nephrol..

[4] Dörner T., Lipsky P.E. (2016). Beyond pan-B-cell-directed therapy — new avenues and insights into the pathogenesis of SLE. Nat. Rev. Rheumatol..

[5] Van de Donk N.W.C.J., Janmaat M.L., Mutis T., Lammerts van Bueren J.J., Ahmadi T., Sasser A.K. (2016). Monoclonal antibodies targeting CD38 in hematological malignancies and beyond. Immunol. Rev..

[6] Lokhorst H.M., Plesner T., Laubach J.P., Nahi H., Gimsing P., Hansson M. (2015). Targeting CD38 with daratumumab monotherapy in multiple myeloma. N. Engl. J. Med..

[7] Dimopoulos M.A., Oriol A., Nahi H., San-Miguel J., Bahlis N.J., Usmani S.Z. (2016). Daratumumab, lenalidomide, and dexamethasone for multiple myeloma. N. Engl. J. Med..

[8] Mateos M.V., Dimopoulos M.A., Cavo M., Suzuki K., Jakubowiak A., Knop S. (2018). Daratumumab plus bortezomib, melphalan, and prednisone for untreated myeloma. N. Engl. J. Med..

[9] Frerichs K.A., Bosman P.W.C., van Velzen J.F., Fraaij P.L.A., Koopmans M.P.G., Rimmelzwaan G.F. (2019). Effect of Daratumumab on Normal Plasma Cells, Polyclonal Immunoglobulin Levels, and Vaccination Responses in Extensively Pre-treated Multiple Myeloma Patients.

[10] Chapuy C.I., Kaufman R.M., Alyea E.P., Connors J.M. (2018). Daratumumab for delayed red-cell engraftment after allogeneic transplantation. N. Engl. J. Med..

[11] Schuetz C., Hoenig M., Moshous D., Weinstock C., Castelle M., Bendavid M. (2018). Daratumumab in life-threatening autoimmune hemolytic anemia following hematopoietic stem cell transplantation. Blood Advances.

[12] Blankestijn M.A., van de Donk N., Sasser K., Knulst A.C., Otten H.G. (2017). Could daratumumab be used to treat severe allergy?. J. Allergy Clin. Immunol..

